# Adult Food Protein-Induced Enterocolitis Syndrome

**DOI:** 10.3389/falgy.2022.889879

**Published:** 2022-05-26

**Authors:** Sara Anvari, Melanie A. Ruffner

**Affiliations:** ^1^Division of Immunology, Allergy, and Retrovirology, Department of Pediatrics, Texas Children's Hospital, Baylor College of Medicine, Houston, TX, United States; ^2^William T. Shearer Center for Human Immunobiology, Texas Children's Hospital, Houston, TX, United States; ^3^Division of Allergy and Immunology, Children's Hospital of Philadelphia, Philadelphia, PA, United States; ^4^Department of Pediatrics, University of Pennsylvania Perelman School of Medicine, Philadelphia, PA, United States

**Keywords:** food protein-induced enterocolitis syndrome, non-IgE allergy, adult food allergy, food allergy, gastrointestinal allergy

## Abstract

Food protein-induced enterocolitis syndrome (FPIES) is a non-IgE, cell-mediated food allergy, commonly diagnosed in infants and young children. In recent years, new-onset adult FPIES has been recognized. The underlying pathogenic mechanism of FPIES has yet to be elucidated, thus disease-specific diagnostic biomarkers have yet to be determined and an oral food challenge (OFC) remains the gold-standard for the diagnosis. Pediatric patients with FPIES classically present with symptoms of delayed, repetitive vomiting approximately 1 to 4 hours following ingestion of a food allergen. However, adults with FPIES have been reported to have a different symptom profile and different food triggers compared to the pediatric FPIES population. The current FPIES diagnostic criteria may not be appropriate for the diagnosis of adult FPIES patients, thus an oral food challenge remains a diagnostic tool. This review provides an overview of the current literature on the clinical presentation, epidemiology, diagnosis, triggers and management of adult FPIES.

## Introduction

Food protein-induced enterocolitis syndrome (FPIES) is a non-IgE, cell-mediated food allergy typically presenting in the first year of life. Patients manifest with symptoms of repetitive, projectile vomiting within 1–4 h of ingesting a food trigger and can also present with pallor and lethargy, and diarrhea may present within 5–10 h. Severe dehydration and hypotension along with metabolic derangements may present in up to 20% of cases (1). The prognosis of FPIES is typically favorable with most children becoming tolerant to their food trigger by 3–5 years of age (1–3).

FPIES is most commonly diagnosed in the pediatric population. However, in recent years, there have been increasing studies reporting FPIES in adults as a heterogenous group of non-IgE mediated food allergic disorders either persisting into or commencing in adulthood. Currently, the 2017 consensus guidelines for the diagnosis of FPIES may not recognize all adult patients with FPIES. This is in part due to the different symptom presentation seen in adults compared to pediatric FPIES patients. Published reports have shown different symptom presentation and triggers in adult-onset FPIES compared to pediatric FPIES. This review will provide an overview of the clinical presentation, epidemiology, diagnosis, triggers, treatment, and disease prognosis with an additional focus on current research gaps and potential future developments for adult-onset FPIES.

## Clinical Presentation

Food protein-induced enterocolitis (FPIES) has been categorized into acute and chronic forms based on the timing of the presentation of symptoms. The chronic form of FPIES was first described in 1978, while the acute form was reported later in 1986 (4, 5). The diagnosis of FPIES has evolved to include clinical criteria (1, 2, 6–8). In 2017, an international consensus guideline for the diagnosis and management of FPIES was published and provided the common major and minor criteria used for the diagnosis of acute FPIES in patients. The major criteria are symptoms of vomiting within 1–4 h after eating the suspected food allergen and the absence of IgE-mediated allergic skin or respiratory symptoms. The minor criteria include two or more episodes of repetitive vomiting after eating the same suspected food, repetitive vomiting 1–4 h after eating a different food, extreme lethargy, marked pallor, the need for emergency department visit, the need for intravenous fluid support, diarrhea within 24 h, hypotension, and/or hypothermia. The manifestations of the symptoms included in these criteria can vary between patients and between episodes of ingestions based on patient age, intrinsic sensitivity, quantity of antigen that was ingested and modifying factors including comorbid conditions like dehydration.

Similarly to acute FPIES, chronic FPIES has classically been defined as a disease limited to infants with an onset during their first few months of life. The major symptoms of chronic FPIES includes diarrhea and failure to thrive. In infants, chronic FPIES has most commonly been described with ingestion of milk or soy. Upon removal of food antigen from the diet, resolution of symptoms can take several days, but patients are at risk of presenting with delayed vomiting and diarrhea consistent with acute FPIES on allergen reintroduction (1, 9).

Both acute and chronic FPIES have traditionally been a disease commonly seen in the pediatric population. However, in adults, both the acute and chronic forms have been recognized and reports have been published (10–14). Thus, the diagnosis of FPIES is no longer limited to a specific age group. In the correct clinical setting, a diagnosis of FPIES should be considered across all ages.

There have been increasing reports of non-IgE mediated food allergy with significant symptom overlap with chronic and acute forms of FPIES seen in children, as well as reports of persistent FPIES through adolescence into adulthood (12, 15). Furthermore, the development of *de novo* delayed non-IgE mediated food allergic symptoms have also been reported to occur in adults (10–13, 16, 17). Patients described multiple, reproducible episodes with gastrointestinal symptoms occurring within 1–4 h following ingestion of the food (11–13, 16–18). Symptom presentation would typically consist of abdominal pain, cramping, diarrhea and/or vomiting consistent with an acute FPIES episode. There have been reports of chronic diarrhea and weight loss consistent with chronic FPIES (13, 14). In, their report on 24 adult FPIES patients, Crespo and colleagues demonstrated that the clinical profile for adult FPIES was more frequently associated with abdominal pain and/or diarrhea. Unlike in infants presenting with FPIES, vomiting was the 3rd most common symptom presentation in adults (19). Skin prick testing with extracts or fresh food are negative, consistent with non-IgE mediated pathophysiology. Symptoms are specifically seen with ingestion of the causative food and elimination diet therapy improved symptoms.

## Epidemiology

The majority of epidemiologic data regarding FPIES has focused on its presentation within children. Population-based cohort studies on FPIES have been reported from Israel, Spain, and Australia. The cumulative incidence of pediatric FPIES was 0.34% in Israel and 0.7% in Spain, and the estimated incidence of FPIES in Australia was reported to be 0.015% per year in infants younger than 2 years (3, 20, 21). In total, these studies suggest that in children, FPIES is more common than had initially been suspected.

Previously published case series examining FPIES have shown that pediatric FPIES has a slight male predominance (3, 22–26). Although limited data exists, when examining gender predominance in adult FPIES, case series to date have 60–90% female patients (Table 1). This is a notable contrast to the pediatric data but should be interpreted with caution given the small numbers of adult patients in case cohorts reported to date.

**Table 1:** Summary of data from case series with adult FPIES patients.

**Study**	**Patients (*n)***	**Female (%)**	**Age at first reaction (y), median IQ_25−75_**	**Reported food(s)**	**Reported symptoms**
Caubet et al. (15)	*	n/a	n/a	Milk, Soy	n/a
Tan et al., (11)	31	77.4	29 (22–45.8)	Shellfish or mollusk (54.8%), egg (16.1%), fish (16.1%), poultry, mushroom, quorn,	Abd. pain (77.4%), vomiting (71%), diarrhea (58.1%)
Gleich et al., (16)	8	87.5	19.5 (14.5–31.5)	Shellfish (100%), mollusk (37.5%), fish, avocado	Abd. pain or cramping (87.5%), nausea/vomiting (75%), diarrhea (62.5%)
Du et al. (10)	20	90	38.5 (min 16, max 67)	shellfish (65%), dairy (20%), wheat (20%), and egg (15%), beef, chicken, mushroom, tomato, cucumber, pepper	Abd. pain or cramping (90%), nausea/vomiting (55%), diarrhea (50%)
Gonzalez-Delgado et al. (12)	25	88	28 (18.5–38)	Shellfish (60%), fish (48%), mollusk (40)	Abd. pain (100%), vomiting (76%), diarrhea (64%), hypothermia or lethargy (28%), loss of consciousness (4%), pallor
Li et al. (18)	19	68.4	28 (min 14, max 68)	shellfish	Vomiting (100%), abd. pain (36.8%), diarrhea (32.1%)

*160 total patients presented in childhood and 5 patients with FPIES to milk or soy did not develop tolerance by age 16. Demographics and symptoms are not separately described for these specific patients.

In 2019, Nowak-Wegrzyn et al published the first lifetime estimated prevalence of FPIES in the United States and in adults (27). This study was based on a population-based survey administered between 2015 and 2016. In adults, the estimated prevalence was about 0.22% with no significant difference in race/ethnicity between patients with and without FPIES. In contrast, children with FPIES were more likely to be of Asian/non-Hispanic ancestry and the estimated prevalence was higher at 0.51%. Both adults and children had increased rates of comorbid IgE-mediated food allergy and other co-morbid atopic conditions. Additional studies have observed that children with FPIES are more likely to have IgE mediated food allergy, atopic dermatitis and other atopic conditions (28).

The presentation of FPIES in adults is less well understood and its current prevalence is unknown. Consensus regarding the diagnostic criteria for adult FPIES is needed both to improve clinical recognition of the disorder and to facilitate accurate studies of FPIES in adults. This is a necessary first step before the true prevalence of FPIES in adults can be assessed.

## Diagnosis

The diagnosis of FPIES is based on clinical history and confirmatory food challenge. Currently there are no known specific biological markers with diagnostic or prognostic value in the care of FPIES patients. Therefore, careful patient history is critical in the diagnosis of FPIES in both adults and children. Clinicians should pay careful attention to the symptoms, timing, and reproducibility of the reactions in relation to eating the specific food. Additionally, symptoms should improve when the food is avoided, and there should be a reproducible set of symptoms that occur with each ingestion of the food. A patient history consistent with FPIES is considered sufficient to make the diagnosis (1).

A notable difference in the presentation between adults and pediatric patients is that there are adult patients reported in several series who do not have vomiting as a feature of their reactions. In a retrospective series of 24 adults, Crespo et al observed diarrhea in most patients (91.7%), followed by crampy abdominal pain (87.5%) and protracted vomiting (75%) (19). Similarly, in case series of adults with non-IgE mediated reactions to shellfish, there were several patients with reactions consisting of abdominal pain or cramping and diarrhea alone (12, 16). In contrast, vomiting is an essential criteria in the acute phenotype of pediatric FPIES and serves as one of the major criteria in the international consensus criteria for FPIES diagnosis (1). Therefore, up to 25% of adults may lack vomiting, which is a currently defined as a major criterion for the diagnosis of FPIES. This suggests that the diagnostic criteria presented in the current international recommendations may ultimately need to be revisited to better reflect what is seen in adults (1).

An oral food challenge is currently the only confirmatory test available for the diagnosis of FPIES. However, due to safety risks, oral food challenges for FPIES are typically reserved for when diagnosis is uncertain or after patients have avoided the food for an interval to determine if tolerance has developed. Oral food challenges that have been performed in adults have followed similar protocols to those that have been published for children (12, 17, 19). However, it was noted that many adult patients declined the offered oral food challenge because of prior severe symptoms (19).

Potential FPIES biomarkers have primarily been studied in the pediatric population and require validation in adult patients. Neutrophilia, thrombocytosis, and leukocytosis during acute FPIES reactions has been observed in pediatric populations (3, 4, 22, 24, 25, 29, 30). This has been reported in a handful of adult patients as well but has not been studied across a large population (17). Other studies have shown that children with FPIES present with normal or mild elevations in CRP when compared to elevated values seen in bacterial gastroenteritis or sepsis (31). It has been suggested that innate pathway dysregulation may be contributing to the development and persistence of FPIES (32, 33). Fever has been reported as a rare presenting feature in pediatric FPIES patients but has not been reported in adult patients (34, 35). At this time, the pathophysiology of FPIES is poorly understood and defined biomarkers have yet to be established. More research is needed in this area, especially in adults.

## Causative Food Triggers

Persistence of FPIES to milk and soy into adolescence and early adulthood was first described in 2014 by Caubet et al. In this study, it was observed that up to 25% of children with FPIES may have low-level detectable specific IgE for the FPIES culprit food, despite having reactions consistent with FPIES and no symptoms to suggest anaphylaxis. There is data to suggest that children with this “atypical” FPIES phenotype may be less likely to develop tolerance over time (15). This was most commonly described in patients with cow's milk FPIES, but has been seen with other antigens.

Crustaceans and mollusks have been frequently reported to cause FPIES in adults (Table 1). This has been commonly reported from several geographic areas including Europe, Canada, and the United States. One notable feature in pediatric FPIES is that there is substantial variability in the most frequent eliciting allergens based on the geographic region (36). To date, there has been less geographic variation observed in the reported causative foods in the adult population when compared to the pediatric population given the predominance of crustacean and mollusk as eliciting allergens. However, it may not be possible to appreciate geographic variability in the adult population at this time given the small numbers of reports.

Although less commonly reported than reactions to seafood, FPIES reactions in adults have been reported to a variety of other foods including cow's milk, hen's egg, finned fishes, beef, poultry and fruits and vegetables (Table 1).

## Treatment and Prognosis

The treatment for FPIES is strict avoidance of the culprit food or foods. Depending on the severity of reaction, administration of intravenous fluids may be necessary. Studies have demonstrated some utility regarding ondansetron in lessening the severity of reactions (37).

There have been reports of patients who develop classic IgE-mediated food allergy following FPIES but this is a less common outcome (38). Case series of adult FPIES patients, particularly with reactions to shellfish, have had negative food-specific IgE or skin testing (16). This is suggestive of a similar non-IgE mediated pathogenesis that is shared between adults and children with FPIES. However, additional studies are needed to understand if detectable food-specific IgE could be used as a prognostic marker to predict pediatric FPIES at risk of persistent symptoms thru adulthood.

Studies examining the natural history of FPIES in infancy/childhood have reported variability in resolution rates for various food triggers. North American studies had variable resolution rates from 20 to 60% for cow's milk, 20–27% for soy, 28–40% for rice, 30–66% for oats, 100% for barley, 67% for vegetables by 3 years of age, while poultry had a resolution rate of 50% by 5 years of age. Resolution rates to the same foods and other foods varied across different regions of the world (36). Among adult FPIES patients, *de novo* reactions in patients who had previously tolerated the food as well as patients who had persistent FPIES through childhood have been reported (12–19). Several authors note that not all patients elect to undergo oral food challenge to determine if the food could be reintroduced and prefer to maintain avoidance. Gonzalez-Delgado et al have published data on adults undergoing oral food challenge with 4 of 25 patients in their cohort ultimately tolerating the triggering food after a period of avoidance (12). Three patients developed tolerance at a mean of 3 years but for one it was at 21 years after the first reaction. Four patients in this cohort had oral challenge reactions with typical symptoms including pain, diarrhea, vomiting and hypothermia. This data suggests there may be a subset of patients who could develop tolerance after a period of avoidance and highlights the need for continued shared decision making regarding risks and benefits of oral challenge in adult patients.

## Discussion

As a rare disease, much remains to be learned about FPIES not only in infants and children, but especially in adults. The majority of FPIES literature to date has primarily demonstrated the predominance of this disease in the pediatric population. However, in the last several years there have been an increasing number of reports that suggest there is a non-IgE mediated, delayed gastrointestinal food allergy that presents in adolescents and adults that closely resembles the presentation of FPIES in children.

The diagnosis of an acute FPIES reaction is dependent on a patient presenting with repetitive vomiting within 1–4 h following allergen ingestion. This presentation is primarily seen in infants and children with FPIES. New evidence suggests that adult FPIES patients may not always present with vomiting and features of their reactions may consist of severe abdominal pain, cramping, and/or diarrhea (12, 16, 19). To better define symptoms associated with adult FPIES, larger clinical studies would further delineate the clinical features in adult FPIES and validate age-specific diagnostic criteria. Additional studies that clarify the spectrum of clinical responses are necessary to determine to what extent adults presenting with this non-IgE food allergy syndrome overlap with children with FPIES, and if unique diagnostic criteria are required for adults with FPIES. Notably, when the 2017 international consensus criteria were published, only a handful of reports describing the development of adult FPIES were published (11, 17). In contrast, there is now more substantial literature, primarily small case series, regarding its non-IgE mediated pathophysiology, unique presentation with gastrointestinal symptoms and potential food triggers.

The true prevalence of FPIES in the adult population is unknown. However, prevalence studies examining childhood FPIES from Israel, Australia, and Spain estimate prevalence between 0.15 and 0.7%, indicating that FPIES in children is less rare than had initially been thought (3, 20, 21, 27). To date, the pathophysiology of FPIES is known to be non-IgE mediated, which is consistent in the reports from the adult population. The specific mechanisms that cause FPIES remain unknown. Longitudinal cohort studies are needed to further examine the innate and adaptive pathways and to identify biomarkers that will provide diagnostic and prognostic information about FPIES in both adults and children. Prognostic data is limited in adult FPIES with most reports suggesting persistence of disease and rare cases of resolution of adult FPIES (12).

In conclusion, there is accumulating clinical evidence that there is a non-IgE mediated gastrointestinal food allergy with delayed onset of gastrointestinal symptoms presenting in adults that mimics both the acute and chronic forms of FPIES seen in pediatric patients. The evidence that adult FPIES exists and can present beyond childhood, suggests that updated guidelines are necessary for the diagnosis, causes, and management of this rare disease in the adult population. There are research gaps (Table 2) in this area not only in diagnosis but also in the understanding the underlying cause of FPIES in both children and adults.

**Table 2:** Research gaps in adult FPIES.

**Patient Presentation**	
•Population-based studies to identify incidence and prevalence
• Are there population differences between adults with *de novo* FPIES and young adults with persistent FPIES from childhood?
**Food Triggers**	
• Frequency of FPIES to multiple foods in adults?
• Are there patterns of cross-reactive allergens in adults with FPIES (i.e.,: as seen with milk and soy in children)?
**Diagnosis**	
• Uniform diagnostic criteria based on adult symptomatology
• Screening studies to identify biomarkers or diagnostic assays
**Management**	
• Optimal oral food challenge protocols
• Study safety and efficacy of antiemetics
• Identify psychosocial impact of FPIES for adult patients
**Prognosis**	
• Prospective studies with food challenges to determine if tolerance occurs
**Etiology**	
• Mechanistic studies of disease pathogenesis and development of allergen tolerance	

## Author Contributions

SA and MR equally contributed to writing the sections of the manuscript. Both authors contributed to manuscript revision, read, and approved the submitted version.

## Conflict of Interest

The authors declare that the research was conducted in the absence of any commercial or financial relationships that could be construed as a potential conflict of interest.

## Publisher's Note

All claims expressed in this article are solely those of the authors and do not necessarily represent those of their affiliated organizations, or those of the publisher, the editors and the reviewers. Any product that may be evaluated in this article, or claim that may be made by its manufacturer, is not guaranteed or endorsed by the publisher.
